# Diet in Rheumatoid Arthritis

**Published:** 1909-10-09

**Authors:** 


### SPECIAL ARTICLE.

### DIET IN RHEUMATOID ARTHRITIS.

We have recently reviewed a book upon "Arthritis Deformans," by Dr. R. Llewellyn Jones,* and we there stated that we thought the author had succeeded in his chief aim, which was to indicate and emphasise the essential distinctness of the diseases—rheumatoid arthritis on the one hand, and osteo-arthritis on the other. In rheumatoid arthritis the joints become deformed, but this is due to the changes in the soft parts only, the bones not being appreciably affected. In osteo-arthritis, on the other hand, the joint deformity depends to a considerable extent upon gross changes in the structure and outline of the ends of the bones themselves. Besides this, with osteo-arthritis the general condition of the patient is, as a rule, good; whilst with rheumatoid arthritis there are nearly always constitutional symptoms, such as loss of weight, loss of appetite, a tendency to pyrexia, a tendency to enlargement of the lymphatic glands, pigmentation of the skin, and various vaso-motor, motor, and sensory-nerve derangements. This being so, it it quite clear that the dietary for rheumatoid arthritis cases needs much more careful supervision than does that of patients suffering from osteo-arthritis.

### The Dietary in Acute Rheumatoid Arthritis.

There is evidence to show that the fundamental condition in rheumatoid arthritis is one of autotoxæmia, and especially when there is oral or nasopharyngeal sepsis. There is a great liability to increase in the gastro-intestinal disturbances so frequently observed in these cases. The toxæmia resulting from the abnormal putrefactive and fermentative processes associated with these conditions indicates that great care should be exercised in dieting these patients. Indeed, as recent researches have shown, in the judicious selection or variation of diet lies our most effectual means of controlling the growth, or modifying the character, of the intestinal flora responsible for the initiation of abnormal putrefactive and fermentative processes, with their remote consequences—chronic toxæmia.

During the acute stages of the disease the nourishment taken should be largely fluid in kind and such as admits of easy digestion and rapid absorption by a febrile patient. Under such circumstances milk will form the staple food. The amount taken will vary with the individual's capacity for digesting it. While some people will find no difficulty in assimilating quantities varying from 2½ pints to 3 pints or more in the 24 hours; others will soon experience nausea, vomiting, and even diarrhœa, from the passage of undigested curds. It is therefore advisable to begin with small amounts given at regular intervals. Where dyspeptic symptoms supervene, these may be minimised by diluting the milk with equal quantities or more of some effervescent alkaline water, or by the addition of three to five grains of citrate of soda to each ounce of milk. Where milk agrees, it should be given up to the limit of the patient's digestive capacity, and its nutritious qualities may be enhanced by the yolk of an egg beaten up in it.

Again, in some instances, patients who object to plain milk will readily partake of one of the prepared foods—Benger's or Mellin's—made up with this vehicle. Plasmon and Sanatogen also deserve mention in the same category.

According to Albu, milk diet constitutes the most effectual means of attaining a comparative degree of intestinal antisepsis. The anti-putrefactive effect of milk is attributable to the lactose it contains, which, under the action of the bacteria of the small intestine, is decomposed into lactic and succinic acids. These acids inhibit the growth of the proteolytic anærobes, which cause putrefaction. These considerations are of great importance when we take into account the frequency with which excessive intestinal putrefaction has been observed in rheumatoid arthritis, especially in view of the constancy with which excess of indican and ethereal sulphates in the urine was noted by Herter in early cases of the affection.

The knowledge that lactic acid bacilli reduced intestinal putrefaction suggested the enhancing of this beneficial action by resorting to a diet consisting largely of curdled or sour milk. Thus, Andrews and Hoke record five cases of rheumatoid arthritis, attended by excessive putrefaction, in which treatment by fermented milk was followed by marked amelioration both of the local and general conditions. Cow's milk fermented by the action of the lactic acid bacillus and brewer's yeast was used. In some instances it was the sole article of diet, as much as three to four quarts daily being ingested.

Tubby, from his own experience, strongly advocates the adoption of this diet in the acute polyarticular form so common in young women, as well as in the more chronic types. Before placing the patient on a strict regimen of soured milk, the presence of an excess of indican and aromatic sulphates in the urine should be established.

As Herschell remarks in his admirable *résumé* on the use of lactic acid ferments in the treatment of disease, it is absolutely necessary that the strictest routine should be observed in the preparation of the soured milk. The natural ferments—the Bulgarian maya and the Egyptian leben—are open to the objection that they contain not only lactic acid bacilli, but other bacteria also, as well as yeasts. The tendency nowadays is to employ pure cultures of lactic acid bacilli. Opinions differ, however, as to whether liquid cultures in tubes or dried cultures in the form of tablets or powders should be utilised for the preparation of the soured milk. Herschell, for example, prefers the latter as more reliable, and in the few instances in which Dr. Jones adopted this mode of treatment his experience accords with

* Bristol: John Wright and Sons, Ltd.

Herschell's as to the superiority of the dried cultures.

The somewhat detailed consideration given to this method of treatment is, he thinks, fully justified in view of the notorious difficulty that attends the treatment of these acute forms. While, reasoning by analogy, the success that has attended this form of treatment in all varieties of auto-intoxication strongly indicates its further employment in rheumatoid arthritis, still the limited number of cases hitherto recorded forbids any dogmatic statements as to its therapeutic value.

Where there exists an insuperable aversion to milk in every form, or a very limited power of assimilating it, there need be no hesitation in supplementing or even replacing it by a liberal allowance of farinaceous foods. Thus, carefully prepared thin oatmeal or barley gruel will often afford a pleasant change, the palatability of which may be increased by the addition of salt or grape sugar, flavoured with nutmeg, lemon-peel, or other agreeable spices. Arrowroot, too, is serviceable, and, like the former, may be prepared with or without milk. Milk puddings, such as rice, sago, tapioca, etc., to which an ounce of ground malt or a tablespoonful of maltine has been added, are often acceptable to the patient, varied with custards and blancmanges made with gelatine.

When, as not infrequently happens in these distressing cases, the patient's progress is slow and unsatisfactory, this strictly lacto-farinaceous regimen must be relaxed in favour of a more mixed diet, and recourse must be had to clear vegetable soups or meat infusions, such as well-made julienne, mutton, veal, and chicken broths, and raw meat-juice. The foregoing may be improved, as Sir William Jenner long since pointed out, by the addition of the expressed juices of fresh vegetables (carrots, parsley, turnips, celery, etc.), or, on the other hand, by adding a tablespoonful of Valentine's meat-juice to each pint.

Should thirst be a prominent symptom, pure water, albumen-water, barley-water, or home-made lemonade form suitable beverages. Hot weak tea, freshly infused, with milk, is often grateful to a febrile patient; eggs may be beaten up with this as a medium. When the vitality is greatly lowered by continuous pyrexia, a little brandy or whisky may prove of considerable value in promoting appetite and assisting digestion.

The caprices of these patients in regard to food are endless, and will tax to the utmost the resources of the physician in his attempts to devise some variation in the monotony of the diet suitable for them at this stage.

While in some instances the course of the pyrexia is continuous, fortunately, in the majority of cases, it is diversified by afebrile intervals. Too much stress cannot be laid on the prime importance of taking advantage of these periods of increased digestive capacity. When they occur, some of the more easily assimilable forms of food should be included in the dietary. Among those permissible in these apyrexial periods are sweetbreads, oysters, lightly boiled fish of the digestible kinds, such as sole and whiting, also pounded and minced meats.

Once convalescence is established, no time should be lost in placing the patient on three good meals a day, with abundance of meat and, if necessary, a reasonable quantity of alcohol. In addition, milk, if possible, should be taken between the meals, and, if such is distasteful in the daytime, it will often be found acceptable either at bedtime or during the night, and may incidentally promote the advent of sleep in a restless subject.

As regards the form of alcohol most suitable for these cases, Dr. Jones has more faith in good stout than any other variety of stimulant. One pint is sufficient within the twenty-four hours, and it may with advantage be distributed over lunch and dinner. He adds that, nauseous and repulsive as it may seem, some patients prefer to take their stout mixed with an equal quantity of milk, and apparently with advantage. Where stout disagrees, or there are conscientious scruples in regard to alcohol, he has found Horlick's malted milk an excellent substitute, and one, moreover, capable of inducing a notable increase of appetite.

In conclusion, he emphasises the importance of not restricting these patients for too long a period to an exclusive milk diet. He is convinced that by so doing their recovery is retarded, a great deal of valuable time being often lost, during which the active progress of the disease might be cut short, and the ultimate degree of crippledom diminished by a more varied regimen.

Doubtless the confusion of these acute forms of rheumatoid arthritis with acute rheumatism is largely responsible for the reluctance displayed by many in placing the patient on a more generous diet. The deplorable results that follow this misconception urgently indicate the desirability of a more timely discrimination between these two joint affections.

In the more slowly progressive forms of the affection it should be taken as a general rule that good nourishing food should be partaken of in any abundance which stops short of derangement of the digestive functions. It must not be overlooked that many of these patients suffer from gastro-intestinal derangements which may either have preceded the rheumatoid affection or have arisen subsequently. In either case it may be taken for granted that no amelioration of the joint condition is to be expected unless these digestive disorders are intelligently and perseveringly treated in accordance with their varied nature. These patients need carefully selected food, and the importance of thorough mastication and regularity in meal-times should be impressed upon them. The desirability of postponing the drinking of fluid to the end of the meal, and then only in small amounts, should be emphasised.

The beneficial effect of rest, both before and after meals, for a period varying from half an hour to an hour, for the purpose of promoting digestion and assimilation, should be taken advantage of. No less important is it that a cheerful atmosphere should be maintained during the actual progress of meals, for it is but too true that "unquiet meals make ill digestions."

The nerve-element in these cases is so pronounced that caution on this point is anything but superfluous, having regard to the inhibitory effects of mental worry and excitement on the secretory mechanisms of the alimentary tract.

With these general considerations we will now enter upon a discussion of the various food-stuffs.

*Animal Foods.*—Owing to the frequent confusion of the more chronic forms of rheumatoid arthritis with gout, marked restriction of, and in some instances complete abstinence from, meat is enjoined, often with disastrous consequences. The still widespread impression that animal food is always undesirable for this class of patients is quite a mistake. The marked and rapid improvement that frequently follows the introduction of a more generous rule in this respect indicates that meat should be allotted a prominent place in any dietary laid down for a rheumatoid patient. The general flabbiness and lack of tone in these patients distinctly suggests their urgent need of this variety of aliment, whose energising and heat-producing qualities are so well ascertained.

Abstention from meat should only be enjoined in the presence of substances in the urine indicative of excessive intestinal putrefaction, meat being, of all articles of food, the chief offender in this respect, because of its marked tendency to undergo putrefaction formation of indol. In the absence of any such indications the following may be taken:—Poultry, fresh game, lamb, mutton, beef, fat bacon, and ham. Fish of all sorts—such as sole, plaice, whiting, turbot, brill, cod, fresh haddock—are permissible. To these, if they agree, may be added salmon, red and grey mullet, herring, and mackerel.

If symptoms of dyspepsia make their appearance, the diet must be modified accordingly. The coarser kinds of fish and meat should be eschewed, and the lighter and more digestible varieties taken in preference. If accompanied by indicanuria and excess of aromatic sulphates, a return must be made temporarily to a strict lacto-farinaceous regimen.

*Farinaceous Foods.*—Some of the subjects of this disease suffer at times from amylaceous dyspepsia, and are unable to digest large amounts of starchy and saccharine foods, as evidenced by considerable discomfort after meals due to flatulent distention. In such cases there should be a reduction temporarily in the amount of this class of food—such as bread, potatoes, milk puddings, etc. In their absence no restriction is called for in this variety of aliment.

*Fats*, such as fresh butter, bone-marrow, fat bacon, and suet in the form of puddings, are highly desirable; while in this category, if it can be taken, cod-liver oil deserves especial mention.

*Vegetables.*—The proportion of vegetable food taken will naturally vary with individual digestive capabilities. As a matter of idiosyncrasy certain articles of this class disagree with certain patients, and such peculiarities must be respected. Short of this, no restriction need be placed upon the patient consuming a moderate quantity of fresh vegetables—such as cauliflower, broccoli, cabbages of all kinds, potatoes, vegetable marrow, sea or Scotch kale, green peas, kidney beans, tomatoes, artichokes, spinach, asparagus, Brussels sprouts, lettuce, radishes, celery, onions, mustard and cress. Where possible vegetables should be given in the form of purées, their digestibility being enhanced by this mode of preparation.

*Salads* should not be partaken of except by those with strong digestive powers.

*Fruits.*—Apples, pears, plums, when of good quality and cooked, are permissible in moderation; while other varieties may be eaten raw when thoroughly ripe, such as peaches, nectarines, and apricots, grapes, fresh figs and dates, also lemons and oranges, when ripe and carefully selected, may be eaten, and the latter are useful for assuaging thirst in these patients when feverish. In addition, walnuts, almonds, and filberts may be partaken of.

*Beverages.*—Tea, coffee, cocoa, and chocolate, when of good quality and properly prepared, are all suitable in moderation; likewise milk, cream, and milk punch. Of alcoholic beverages, malt liquors are the most desirable. Where these disagree, or are unacceptable, a good red wine—such as Burgundy or a sound claret—may be substituted. Whisky or brandy diluted with Apollinaris or other mineral water may be taken instead if preferred; but, whatever the variety of the stimulant chosen, it should be only taken under the direction of the physician, its amount and time of ingestion being strictly fixed.

In the localised forms, more especially that form of osteo-arthritis in the knees which attacks women at the menopause, modification of diet is often called for. The onset of the disease at this period frequently coincides with a notable increase in the patient's body weight, and in his opinion this, through its mechanical action, promotes the advance of the disease. It must be remembered that in this affection, as opposed to the rheumatoid arthritis, bony nodes are of constant occurrence, and by reason of their presence the joint is in an extremely irritable condition in the early stages of the malady. Any notable increase in the patient's weight, therefore, is calculated to aggravate the irritation produced by these bony outgrowths. Goldthwait and Painter have shown that localised villous arthritis, not to mention recurring synovitis, may result in these cases from irritation caused by bony spurs.

In this particular type of the disease, Dr. Jones has found it of the greatest value to reduce, or even abolish for a time, the carbohydrate and fatty constituents of the diet, and also all malt liquors. Indeed, alcohol, as far as possible, should be rigidly excluded, or only a very moderate quantity of good whisky, or a light wine—such as Moselle—should be permitted. All forms of meat, poultry, fish, and game, may be partaken of freely. All vegetables, also, with the exception of the roots and tubers such as potatoes, etc., may be eaten. Bread should be largely reduced in quantity, and only eaten after being thoroughly "torrified." Sugar should be prohibited, and saccharin, or saxin, used as a sweetening agent. Milk, cream, and butter should be very strictly limited. Hot water may with advantage be taken either an hour before or two hours after meals for its flushing effects. Should the urine become concentrated and contain a large amount of urates while on this proteid diet, barley-water or some alkaline mineral water should be freely partaken of. At meal-times fluid should be strictly limited.

